# Clinical, histopathological characteristics and malignant transformation of proliferative verrucous leukoplakia with 36 patients: a retrospective longitudinal study

**DOI:** 10.1186/s12903-024-04360-0

**Published:** 2024-05-30

**Authors:** Chang Zhang, Qingying Lan, Pan Wei, Yan Gao, Jianyun Zhang, Hong Hua

**Affiliations:** 1 Department of Oral Medicine, Peking University School and Hospital of Stomatology & National Center of Stomatology & National Clinical Research Center for Oral Diseases & National Engineering Research Center of Oral Biomaterials and Digital Medical Devices & Beijing Key Laboratory of Digital Stomatology & NHC Key Laboratory of Digital Stomatology & NMPA Key Laboratory for Dental Materials, No. 22, Zhongguancun South Avenue, Haidian, Beijing, 100081 China; 2 Department of Oral Pathology, Peking University School and Hospital of Stomatology & National Center of Stomatology & National Clinical Research Center for Oral Diseases & National Engineering Research Center of Oral Biomaterials and Digital Medical Devices & Beijing Key Laboratory of Digital Stomatology & NHC Key Laboratory of Digital Stomatology & NMPA Key Laboratory for Dental Materials, No. 22, Zhongguancun South Avenue, Haidian, Beijing, 100081 China; 3 Research Unit of Precision Pathologic Diagnosis in Tumors of the Oral and Maxillofacial Regions, Chinese Academy of Medical Sciences (2019RU034), Beijing, China

**Keywords:** Oral cavity, Malignant transformation, Proliferative verrucous leukoplakia, Oral epithelial dysplasia

## Abstract

**Background:**

Proliferative verrucous leukoplakia (PVL), distinguished by its malignant transformation rate of 43.87% to 65.8%, stands as the oral potentially malignant disorder with the highest propensity for malignancy. PVL is marked by distinctive heterogeneity regarding the clinical or histopathological characteristics as well as prognostic factors pertinent to this condition. The purpose of this study is to compile and assess the clinicopathological features, malignant transformation, and associated risk factors in patients diagnosed with PVL.

**Methods:**

This study is a hospital-based retrospective longitudinal study of 36 patients diagnosed with PVL from 2013 to 2023. We conducted complete clinical and histopathological evaluations of the patients.

**Results:**

The cohort comprised 16 males and 20 females, yielding a male-to-female ratio of 1:1.25. The follow-up period ranged from 8 to 125 months, with an average of 47.50 months. The most common clinical type of lesion was the verrucous form (58.33%), and the gingiva was the most common site (44.44%). Each patient had between 2 to 7 lesions, averaging 3.36 per patient. During the follow-up period, twelve patients (33.3%) developed oral cancer, with an average time to malignant transformation of 35.75 months. Kaplan–Meier survival analysis indicated that patients with complaints of pain, roughness, or a rough sensation, with diabetes, and the presence of cytologic atypia histologically showed a higher risk of malignant transformation (*p* < 0.05). In this study, the rate of malignant transformation in the treatment group (5/23) was lower than that in the untreated group (7/13), however, no statistically significant difference (*p* = 0.05).

**Conclusion:**

The main complaints of pain, roughness, or foreign body sensation, coupled with cytologic atypia histologically are indicative of an increased risk of malignant transformation in PVL. Further research is needed to elucidate the influence of these clinicopathological parameters on the malignant progression of PVL.

## Introduction

The global incidence of oral cancer is on the rise, with approximately 377,713 new cases reported in 2020. Oral squamous cell carcinoma (OSCC), the most common type of oral cancer, exhibits a 5-year survival rate of less than 50% in patients diagnosed at advanced stages. This survival rate drops to below 30% in cases with extensive lymph node metastasis [1–4]. Oral potentially malignant disorders (OPMDs) represent a group of oral mucosal lesions that may undergo malignant transformation(MT) to oral cancer. Oral leukoplakia (OLK) is the main type of OPMDs with a malignancy transformation rate of 9.5%—9.8% [5]. Proliferative verrucous leukoplakia (PVL) is considered a rare and distinct type of leukoplakia characterized by an unknown etiology, characterized by its progressive, multifocal nature, predominantly found in elderly women. It has the highest rate of MT among all OPMD [6–8]. Recent meta-analyses have shown that the overall malignancy rate of PVL can range from 43.87% to 65.8% with some studies reporting rates as high as 100% [9–14]. The studies into clinical and pathological features of PVL, along with its risk of MT, are crucial for its in-depth understanding, early precise diagnosis, and treatment. Additionally, it contributes to improving the prognosis and survival rates of patients with oral cancer.

The concept of "Proliferative verrucous leukoplakia" was first proposed by Hansen et al. in 1985, namely, PVL is a special leukoplakia that is slow-growing, persistent, irreversible, and resistant to every kind of therapy, which can start from simple hyperkeratosis into multifocal lesions and evolve into a verrucous carcinoma and squamous cell carcinoma [15]. Saito et al. suggested that PVL might develop with homogeneous leukoplakias, and some studies proposed the term "proliferative multifocal leukoplakia" [16, 17]. The diagnostic criteria of PVL have been proposed by different research teams. The diagnostic criteria proposed in 2010 and 2018 emphasize the characteristics of a large range of lesions, female patients, and a long course of the disease [12, 13]. The latest diagnostic criteria for PVL proposed by Gonzalez-Moles et al. in 2021 were multifocal white plaques, which have expanded throughout its evolution, persistent and resistant to treatment. Most of the patients were diagnosed at an age older than 60, with a very high risk of PVL developing into oral cancer and a lack of special histopathological findings [18].

Currently, clinical research on PVL is relatively rare. The diversity in selection criteria among research results in high heterogeneity in data and conclusions [9, 11]. There is significant heterogeneity in clinical, pathological, and prognostic aspects. Only a few studies have described the histopathological characteristics in detail. Based on the above findings, the study aims to summarize the clinical and histopathological characteristics and prognosis of PVL and to provide a theoretical basis for the early intervention of PVL.

## Materials and methods

### Study population

We screened the patients with oral leukoplakia whose lesions showed verrucous form in clinical type or verrucous morphology in histology from 2013 to 2023. The observation period started on the patient's first visit to the hospital and ended on December 1, 2023. We retrieved the patient's medical records and updated them from December 2023 to January 2024. The diagnosis criteria proposed by Gonzalez-Moles et al. in 2021 were used [18]. Oral leukoplakia with multifocal and progressive characteristics will be diagnosed as PVL and other diseases excluded by histopathological biopsy. If the multifocal and progressive characteristics cannot be evaluated due to the lack of corresponding data, they will be recorded as invalid data. The inclusion criteria for patient enrollment were as follows: (1) patients were required to have undergone at least one biopsy and to have had two or more examinations during the follow-up period; (2) a definitive diagnosis of leukoplakia was made, with lesions exhibiting a verrucous form clinically or verrucous morphology histologically. The exclusion criteria were defined as: (1) an initial biopsy revealing oral cancer; (2) a diagnosis of chronic hyperplastic candidiasis; (3) absence of follow-up data or a follow-up period of less than six months.

### Data extraction

Two authors (ZC and LQY) extracted information from the patient's medical record. We documented the clinical characteristics of the patients, including the gender, age at initial diagnosis, onset time, clinical diagnosis, clinical type, lesion size, lesion number, location of the initial biopsy, locations involved during follow-up, duration of follow-up, therapeutic interventions the systemic conditions (such as hypertension, type 2 diabetes mellitus et al.), consumption of tobacco and alcohol, as well as the occurrence of malignant transformation over time and its associated locations. According to the classification standard of epithelial dysplasia and the description of PVL pathological characteristics proposed in the 5th edition of the World Health Organization head and neck tumor classification [19], two pathologists (GY and ZJY) respectively interpreted the hematoxylin–eosin stained paraffin sections of the previous biopsy of all PVL patients and classified the degree of epithelial dysplasia as mild dysplasia, moderate dysplasia, and severe. Meantime, they recorded the following features: (1) premature keratinization; (2) sharp lateral margins; (3) skip keratoses; (4) cytologic atypia; (5) typical architectural feature: corrugated hyperortho or parakeratotic lesions with a verucco-papillary architecture; (6) an exophytic or endophytic growth pattern showing a verrucous, nodular, or bulky; (7)a band-like lymphohistiocytic infiltrate. ﻿When opinions differed, the two pathologists discussed to achieve consensus﻿.

### Statistical analysis

The statistical analysis in this study was carried out using IBM SPSS 26.0 software (SPSS, Chicago, IL, USA). A descriptive analysis of clinical and pathological factors was carried out. Continuous variables are represented by means with SD or medians with ranges, and categorical variables are represented by the frequency and proportion in the population. To investigate the association between risk factors and MT, Student's t test or Mann–Whitney U test was applied in the analysis of continuous variables, and Pearson's chi square test or Fisher's exact test for categorical variables. All P-values in this study were tested bilaterally, with a significance level of *p* < 0.05 and a confidence interval of 95%. We used stratified analysis in follow-up time. Furthermore, this study utilized Kaplan Meier survival analysis to construct survival curves, with the survival time defined as the time from first diagnosis and cancer development (non-censored data) or until the end of patient follow-up (censored data).

## Results

### Screening of PVL patients

**Fig. 1:**
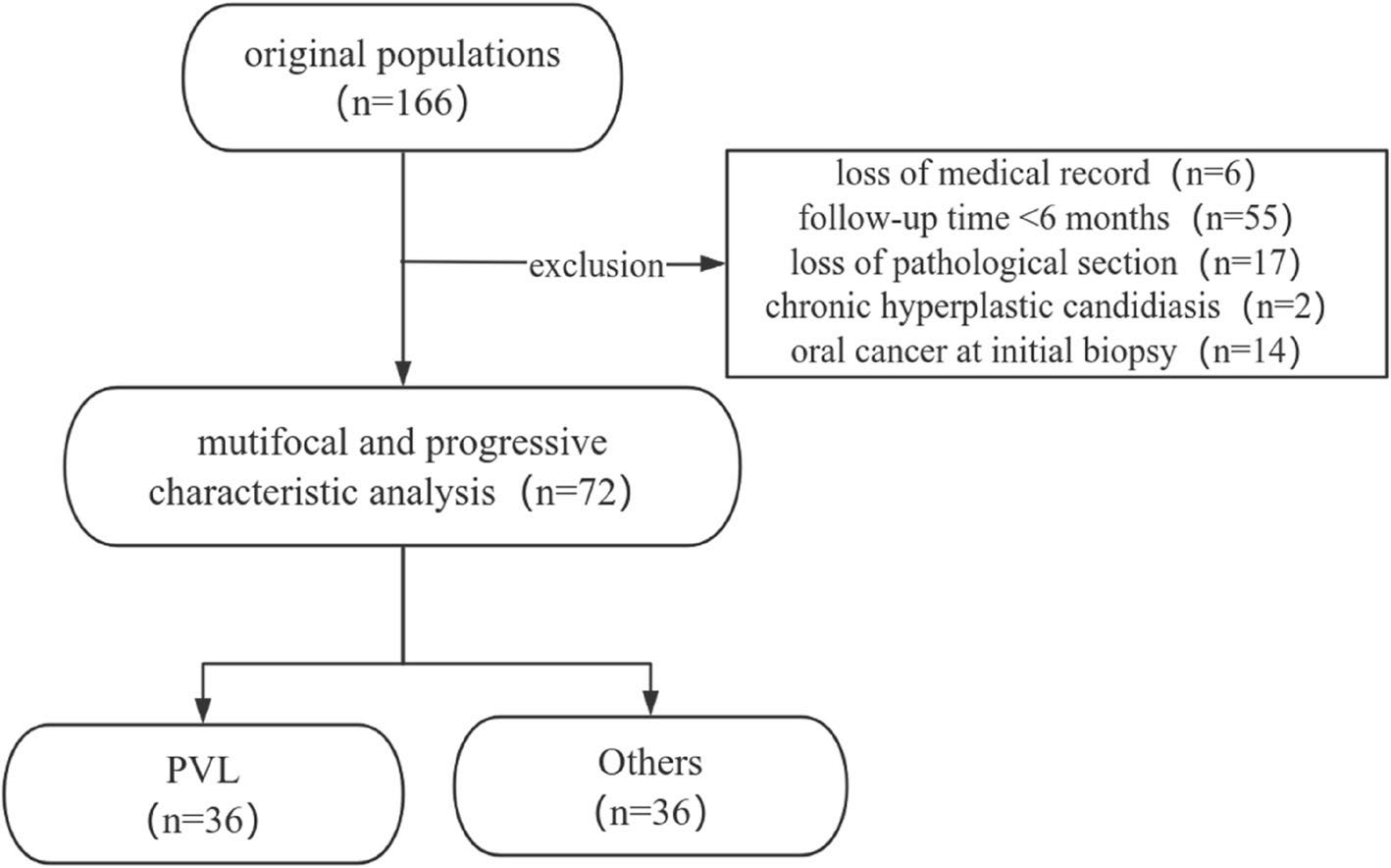
The flow chart of the patients screening

Between 2013 and 2023, 166 patients presented with verrucous form clinically or displaying verrucous morphology histologically, initially diagnosed with oral leukoplakia in our department. After applying the inclusion and exclusion criteria, 94 patients were ultimately excluded from the study, leaving a cohort of 72 patients in whom multifocal and progressive characteristics were examined. A total of 36 patients were found to meet the PVL diagnostic criteria (Fig. 1).

### Clinical characteristics

**Fig. 2:**
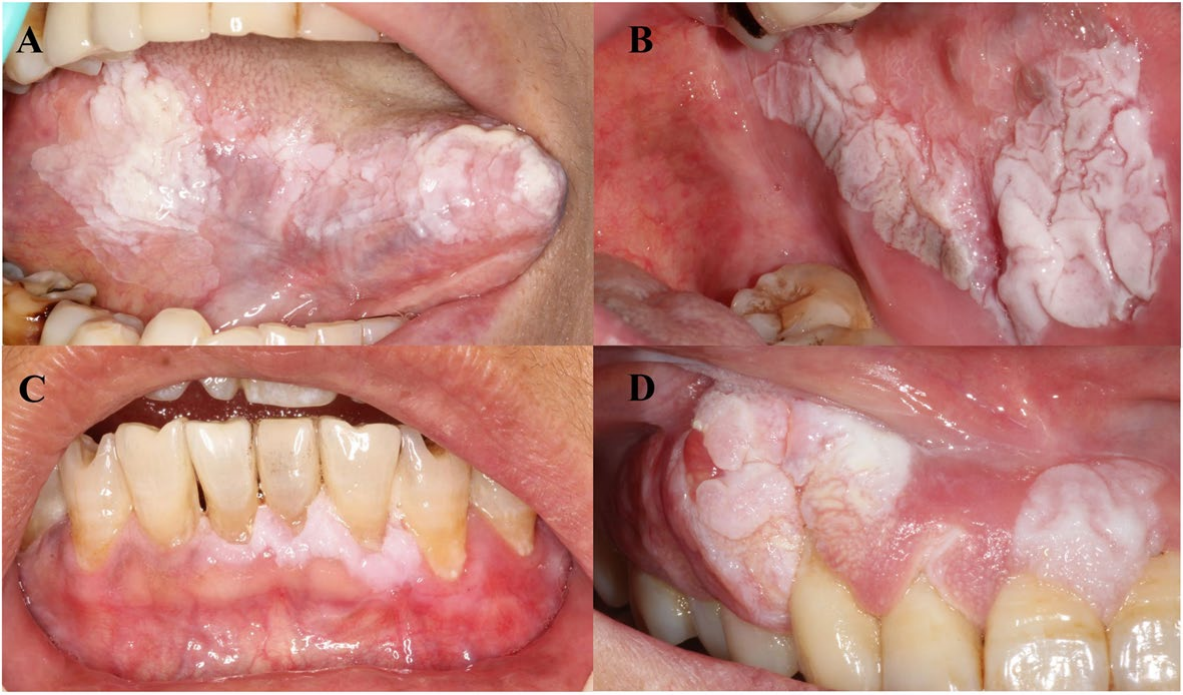
Oral clinical manifestations in patients with PVL: **A** a large area of white verrucous lesion with a raised and irregular surface texture is predominantly localized along the right lateral border of the tongue; **B** the widespread area of white, fissured plaque on the left buccal mucosa; **C** a thick white lesions spread along the gingival margin; **D** the white warty lesion with localized erythematous appearance involving the upper maxillary gingiva

Among the 36 patients diagnosed with PVL, 20 were females and 16 were males, yielding a female-to-male ratio of 1:1.25, with an average age of 62.972 ± 11.949 years at initial diagnosis. The average age of female patients at initial diagnosis was 65.00 ± 8.49 years, which was higher than that of males. All female patients were over the age of 50 at the time of their initial diagnosis, with 65% being 60 years old or older. 66.67% (24/36) of patients seek medical assistance due to the discovery of oral white lesions without discomfort. Among the initial biopsy sites, the gingiva was the most common (16/36), followed by the buccal mucosa(9/36) and the tongue(7/36). The biopsy sites of the tongue are all distributed along the lingual margin and abdomen. In addition, two patients had biopsy sites located on the floor of the mouth, and two patients had biopsy sites located in the palate and corner of the mouth, respectively. The average lesion size of the patient is 2.54 ± 1.50 cm. At the initial diagnosis, the most common manifestation of lesions was verrucous form, accounting for 58.33%, while 8 patients showed homogeneous type, accounting for 22.22%. Additionally, 8.33% patients showed nodular leukoplakia, and 11.11% patients showed speckled leukoplakia. The clinical manifestations indicate a protean nature (Fig. 2). Of the patients, 19.44% (7/36) had a history of drinking, while 27.78% (10/36) had a history of smoking. Among males, there were significantly more patients with a history of smoking and drinking than females, with statistical differences (*p* < 0.05), as shown in Table 1.

**Table 1:** Demographic characteristics and clinical features of the patients with PVL

	Total *N* = 36	Female *N* = 20	Male *N* = 16	*p* value
Age(Years)				
Mean(SD)	62.97(11.95)	65.00(8.49)	60.44(15.15)	^t^0.293
Range	26–82	54–82	26–76	
Complaint				
White lesions(n,%)	24(66.67)	13(65.00)	11(68.75)	^a^1.000
Discomfort(n,%)	12(33.33)	7(35.00)	5(31.25)	
Location first biopsy				
Gingiva(n,%)	16(44.44)	12(60.00)	4(25.00)	^b^0.107
Tongue(n,%)	7(19.44)	2(10.00)	5(31.25)	
Buccal(n,%)	9(25.00)	5(25.00)	4(25.00)	
Others(n,%)	4(11.11)	1(5.00)	3(18.75)	
Area(cm^2^)				
< 2(n,%)	14(38.89)	9(45.00)	5(31.25)	^b^0.472
2–4(n,%)	8(22.22)	3(15.00)	5(31.25)	
≥ 4(n,%)	14(38.89)	8(40.00)	6(37.50)	
Diameter(cm)				
≥ 3 cm(n,%)	15(41.67)	9(45.00)	6(37.50)	^a^0.741
< 3(n,%)	21(58.33)	11(55.00)	10(62.50)	
Clinical form				
Homogeneous(n,%)	8(22.22)	4(20.00)	4(25.00)	^a^0.078
Verrucous(n,%)	21(58.33)	9(45.00)	12(75.00)	
Nodular(n,%)	4(11.11)	4(20.00)	0(0)	
Speckled(n,%)	3(8.33)	3(15.00)	0(0)	
Alcohol consumption				
Yes(n,%)	7(19.44)	0(0)	7(43.75)	^a^0.001
Never(n,%)	29(80.56)	20(100.00)	9(56.25)	
Tobacco consumption				
Yes(n,%)	10(27.78)	1(5.00)	9(56.25)	^a^0.002
Never(n,%)	26(72.22)	19(95.00)	7(43.75)	
Dysplasia				
No(n,%)	8(22.22)	3(15.00)	5(31.25)	^b^0.365
Mild(n,%)	16(44.44)	8(40.00)	8(50.00)	
Moderate(n,%)	7(19.44)	5(25.00)	2(12.50)	
Severe(n,%)	5(13.89)	4(20.00)	1(6.25)	
Onset time (months)				
Median(range)	24(1–144)	27(1–144)	21(1–84)	^m^0.083
Follow-up time(months)				
Median(range)	47.50(8–125)	48.50(8–122)	29.50(9–125)	^m^0.435
Total lesions				
Average (SD)	3.36(1.44)	3.80(1.67)	2.81(0.83)	^t^0.023

SD Standard deviation, Discomfort: pain, roughness, or foreign body sensation at the initial visit, ^a^Fisher’s exact test, ^b^Pearson Chi-square test, ^t^Student's t test, ^m^MannWhitney U test

The onset time of PVL patients is from 1 to 144 months, with a median of 24 months. The average follow-up time is 47.50 months, with a minimum of 8 months and a maximum of 125 months. We found 69.44% (25/36) of patients showed gingival involvement, 50% (18/36) of patients showed buccal involvement, and 36.11% (13/36) of patients presented with lingual margin and abdominal involvement in the course of the disease. The average number of oral lesions in 36 patients with PVL is 3.36. We performed a stratified analysis of the clinical characteristics between male and female patients. The number of lesions in females is 3.80 ± 1.67, which is greater than that in males(2.81 ± 0.83) with a statistical difference (*p* = 0.023) (Table 1).

### Histopathological characteristics

**Fig. 3:**
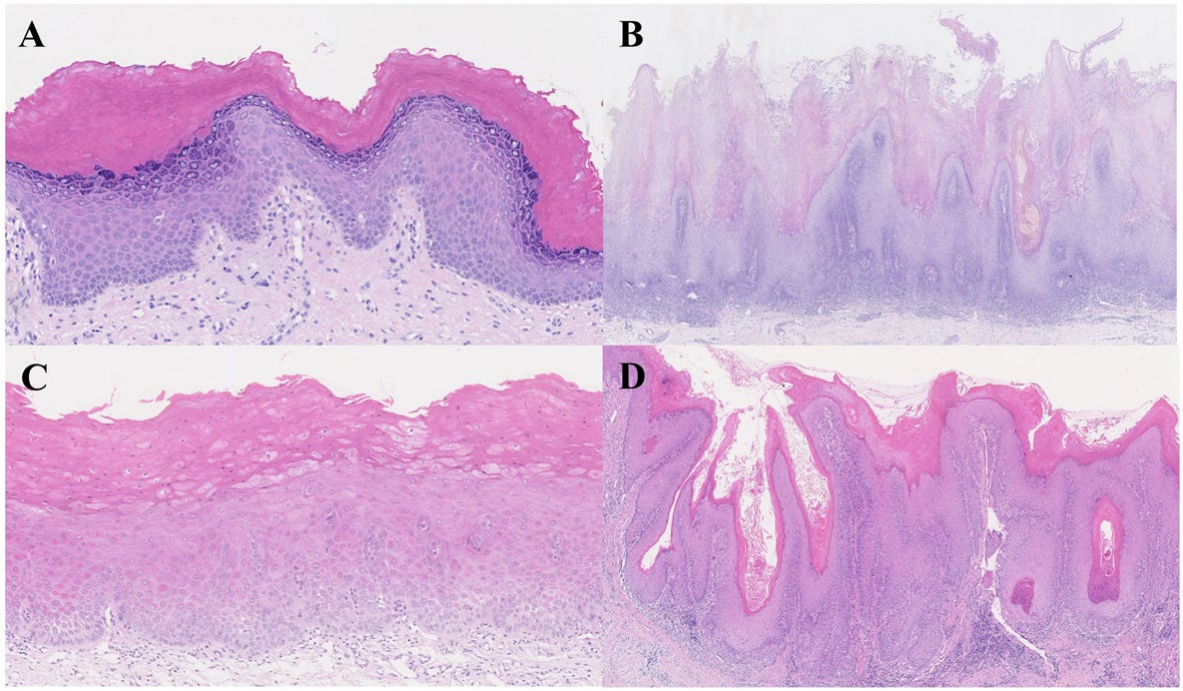
The histopathological characteristics in patients with patients with PVL: **A** marked orthohyperkeratosis and epithelial corrugation without dysplasia (H&E stain, original magnification × 200); **B** corrugated hyperorthokeratotic lesions with a verucco-papillary architecture with band-like lymphohistiocytic infiltrate (H&E stain, original magnification × 40); **C** epithelial proliferation with premature keratinization covered by sharp lateral margins (H&E stain, original magnification × 200); **D** the epithelium is bulky and thickened with cytologic atypia(H&E stain, original magnification × 80)

Two pathologists independently assessed the degree of epithelial dysplasia using the 2022 diagnostic criteria. The interpretation results had a consistency rate of 77.78%, and the linear weighted Kappa value of 0.783 indicated substantial consistency [20]. Among the 36 patients, 22.22% had no dysplasia, 44.44% had mild dysplasia, 19.44% had moderate dysplasia, and 13.89% had severe dysplasia. 13.89% (5/36) of patients showed skip keratoses on initial biopsy. Band-like lymphohistiocytic changes occurred in 61.11% (22/36) of patients. Premature keratinization, cytologic atypia, and architectural features such as verrucous, nodular, or bulky patterns were observed more frequently and showed a statistically significant correlation with the presence of moderate to severe dysplasia (Table 2; Fig. 3).

**Table 2:** Histological features in the patients of the first biopsy

Dysplasia	No *N* = 8	Mild *N* = 16	Moderate *N* = 7	Severe *N* = 5	*p*	*p* (Moderate , Severe)
Premature keratinization(n)	1	3	3	4	0.038	0.020
Sharp lateral margins(n)	2	7	3	1	0.743	1.000
Skip keratoses(n)	1	3	1	0	0.911	0.646
Cytologic atypia(n)	0	13	6	4	< 0.001	0.143
Typical architectural feature(n)	8	16	7	4	0.139	0.333
Verrucous, nodular, or bulky architecture(n)	0	4	4	5	0.001	0.001
Band-like lymphohistiocytic infiltrate(n)	3	11	4	4	0.416	0.727

### Treatment

**Fig. 4:**
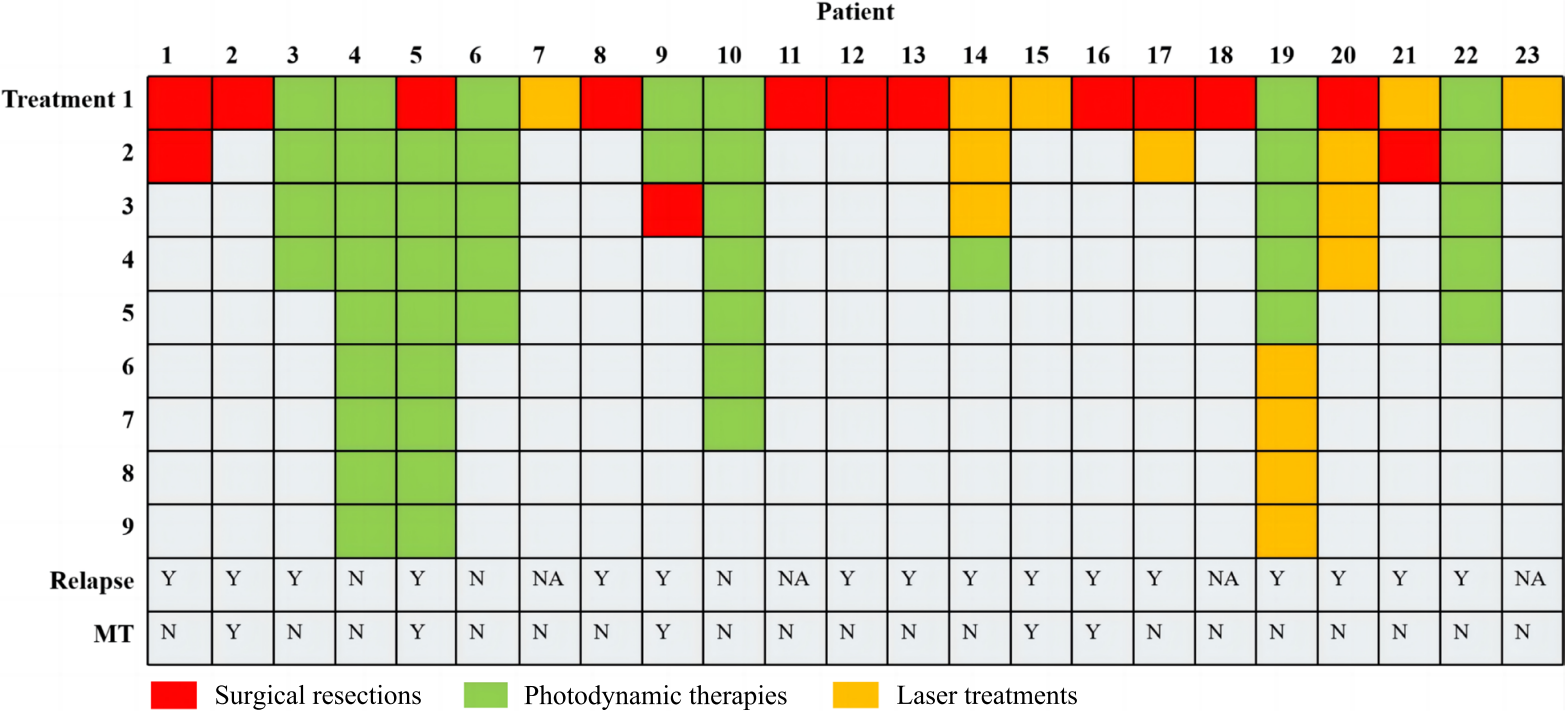
A heat-map-like diagram illustrating the therapeutic interventions administered to the 23 patients, categorized by treatment sessions. Each cell represents a specific intervention and corresponds to a patient in the queue by columns and the respective treatment session by rows (Y: Yes, N: No, NA: Not available)

63.89% (23/36) of patients underwent therapeutic interventions. 36.11% (13/36) of patients underwent 1–2 surgical resections. Approximately 25% of the patients received photodynamic therapy, with the treatment frequency spanning from one to nine sessions and a mean of 5.11 treatments. Furthermore, a subset of eight patients was administered semiconductor laser therapy, ranging from one to four sessions, with an average of 1.88 treatments. Notably, six patients underwent a combination of both therapeutic modalities. Among the 23 patients, only 3 did not relapse, and 3 could not be evaluated due to a lack of necessary data (Fig. 4). Of the 23 patients undergoing treatment, MT occurred in 5 patients, with a median time to MT of 31 months (range: 10–62 months). Conversely, among the 13 patients who received no therapy, 7 patients developed MT, with a median time to MT of 27 months (range: 2–122 months).

### Risk factors of MT

Among 36 patients with PVL, 12 patients developed malignant transformation, with a median interval from initial diagnosis to MT of 29 months, a minimum of 2 months, and a maximum of 122 months. Ten patients were diagnosed with squamous cell carcinoma, while two were identified with verrucous carcinoma. Among the 23 patients who received treatment, 21.74% (5/23) developed MT. In the cohort of 13 patients who received no intervention, 53.85% (7/13) experienced MT. No statistically significant correlation was observed between treatment and MT(*p* = 0.050).

The sites included the gingiva (3/12), the tongue (3/12), the buccal mucosa (3/12), and the upper lip(1/12). Two patients exhibited two sites with MT involvement. 91.67% (11/12) of patients had MT development sites encompassing their initial biopsy locations. One of these individuals showed MT at the corner of the mouth, in addition to the tongue, and another showed MT in the buccal mucosa, in addition to the gingiva. Despite receiving an initial gingiva biopsy, one patient developed MT on the upper lip. We conducted an analysis of the association between different clinical features and MT. No statistical differences were observed regarding gender, age, initial biopsy site, extent of the lesion, hypertension, diabetes, candidiasis, smoking history, and alcohol consumption (Table 3). One-third of patients experience discomfort such as pain, roughness, or foreign body sensation at the initial visit. The complaints of discomfort associated with the lesions showed a correlation with the risk of MT, which was statistically significant (*p* = 0.007).

**Table 3:** Malignant transformation and associated risk factors

	Total *N* = 36	MT *N* = 12	No MT *N* = 24	*p*
Sex				^a^0.157
Female(n,%)	20(55.56)	9(75.00)	11(45.83)	
Male(n,%)	16(44.44)	3(25.00)	13(54.17)	
Age				
Mean(SD)	62.97(11.95)	62.17(13.47)	62.38(11.25)	^t^0.779
≥ 60(n,%)	23(63.89)	7(58.33)	16(66.67)	^a^0.868
50–60(n,%)	7(19.44)	4(33.33)	5(20.83)	
< 50(n,%)	6(16.67)	1(8.33)	3(12.50)	
Complaint				^a^0.007
White lesions(n,%)	24(66.67)	4(33.33)	20(83.33)	
Discomfort(n,%)	12(33.33)	8(66.67)	4(16.67)	
Diagnosis with LM				^a^0.126
No (n,%)	27(75.00)	7(58.33)	20(83.33)	
Yes(n,%)	9(25.00)	5(41.67)	4(16.67)	
Clinical				^a^0.224
Homogeneous(n,%)	8(22.22)	1(8.33)	7(29.17)	
Non(n,%)	28(77.77)	11(91.67)	17(70.83)	
Location				^b^0.332
Gingival(n,%)	16(44.44)	5(41.67)	11(45.83)	
Tongue(n,%)	7(19.44)	4(33.33)	3(12.50)	
Buccal(n,%)	9(25.00)	3(25.00)	6(25.00)	
Others(n,%)	4(11.11)	0(0)	4(16.67)	
Area(cm^2^)				^m^0.481
Median	2.325	3.2	2.20	^b^0.852
< 2(n,%)	14(38.89)	5(41.67)	9(37.50)	
2–4(n,%)	8(22.22)	2(16.67)	6(25.00)	
≥ 4(n,%)	14(38.89)	5(41.67)	9(37.50)	
Diameter(cm)				
Median	2.10	2.50	2.10	^m^0.585
< 3(n,%)	21(58.33)	6(50.00)	15(62.5)	^b^0.473
≥ 3(n,%)	15(41.67)	6(50.00)	9(37.5)	
Total lesions				
Average (SD)	3.36(1.44)	4.00(1.71)	3.04(1.20)	^t^0.058
Dysplasia				^a^0.636
No(n,%)	8(22.22)	1(8.33)	7(29.17)	
Mild(n,%)	16(44.44)	6(50.00)	10(41.67)	
Moderate(n,%)	7(19.44)	3(25.00)	4(16.67)	
Severe(n,%)	5(13.89)	2(16.67)	3(12.20)	
Follow-up time(months)				
Median	40.5	65.5	24	^m^0.005
< 29(n,%)	15(41.67)	0(0)	15(62.50)	^a^ < 0.001
≥ 29(n,%)	21(58.33)	12(100.00)	9(37.50)	
Candidiasis				^a^0.149
Yes(n,%)	6(16.67)	4(33.33)	2(8.33)	
No(n,%)	30(83.33)	8(66.67)	22(91.67)	
Hypertension				^a^0.446
Yes(n,%)	11(30.56)	5(41.67)	6(25.00)	
No(n,%)	25(69.44)	7(58.33)	18(75.00)	
Diabetes mellitus				^b^0.307
Yes(n,%)	5(13.89)	3(25.00)	2(8.33)	
No(n,%)	31(86.11)	9(75.00)	22(91.67)	
Tobacco consumption				^a^0.438
Yes(n,%)	10(27.78)	2(16.67)	8(33.33)	
Never(n,%)	26(72.22)	10(83.33)	16(66.67)	
Alcohol consumption				^a^1.000
Yes(n,%)	7(19.44)	2(16.67)	5(20.83)	
Never(n,%)	29(80.56)	10(83.33)	19(79.17)	

SD Standard deviation, LM Lichenoid morphology, Discomfort: pain, roughness, or foreign body sensation at the initial visit, ^a^Fisher’s exact test, ^b^Pearson Chi-square test, ^t^ Student's t test, ^m^Mann–Whitney U test

Regarding the initial biopsy pathology, 12.50% (1/8) of patients without dysplasia developed malignant transformations, while among the 28 patients with epithelial dysplasia, 11 progressed to malignancy. MT developed in 37.50% (6/16) of patients with mild dysplasia, and the corresponding rates for moderate and severe dysplasia were 42.86% (3/7) and 40.00% (2/5), respectively. As the degree of epithelial dysplasia increases, there is an observed rise tendency in the risk of malignant transformation (*p* = 0.636). Among the 24 patients without MT, 11 experienced two biopsies. Five patients showed progression in the degree of dysplasia compared to the initial biopsy. Four individuals transitioned from having no epithelial dysplasia to various degrees of dysplasia, and one progressed from mild to severe dysplasia.

Within the pathological characteristics, 91.67% (11/12) of patients with MT and 50% (12/24) of patients without MT presented cytologic atypia, indicating a statistically significant correlation with MT (*p* = 0.025). Among patients who developed MT, 50% (6/12) showed significantly proliferative verrucous structures, surpassing the 29.17% (7/24) observed in patients without MT. The frequency of skip keratoses was 16.67% (2/12) in patients who experienced MT, marginally exceeding the 12.50%(2/12) showed in those without MT (Table 4).

**Table 4:** Malignant transformation and histological features

	MT *N* = 12	No MT *N* = 24	*p*
Premature keratinization			^a^0.715
Yes	3(25.00)	8(33.33)	
No	9(75.00)	16(66.67)	
Sharp lateral margins			^a^0.468
Yes	3(25.00)	10(41.67)	
No	9(75.00)	14(58.33)	
Skip keratoses			^a^1.000
Yes	2(16.67)	3(12.50)	
No	10(83.33)	21(87.50)	
Cytologic atypia			^a^0.025
Yes	11(91.67)	12(50.00)	
No	1(8.33)	12(50.00)	
Typical architectural feature			^a^0.333
Yes	11(91.67)	24(100.00)	
No	1(8.33)	0(0)	
Verrucous, nodular, or bulky architecture			^b^0.220
Yes	6(50.00)	7(29.17)	
No	6(50.00)	17(70.83)	
Band-like lymphohistiocytic infiltrate			^b^0.809
Yes	7(58.33)	15(62.50)	
No	5(41.67)	9(37.50)	

^a^Fisher Accurate Chi-square test, and ^b^Pearson Chi-square test

The follow-up duration was found to be significantly correlated with the risk of MT, with statistical significance (*p* = 0.005). Based on the median time to MT, we discovered that a follow-up duration of ≥ 29 months was significantly correlated with MT, with a statistically significant difference (*p* < 0.001). Among patients with a follow-up duration of ≥ 29 months, the malignant transformation rate could reach 57.14% (12/21), while none of the patients with a follow-up duration of less than 29 months had developed cancer by their last examination. We conducted a stratified analysis in patients with a follow-up duration of ≥ 29 months and found that the presence of epithelial dysplasia was significantly correlated with the risk of malignant transformation (*p* = 0.016), and cytologic atypia was also significantly associated with an increased risk of MT (*p* = 0.002). Moreover, in stratified analysis, symptoms such as pain, roughness, or foreign body sensation did not exhibit a statistically significant correlation with the risk of MT (*p* = 0.080).

**Fig. 5:**
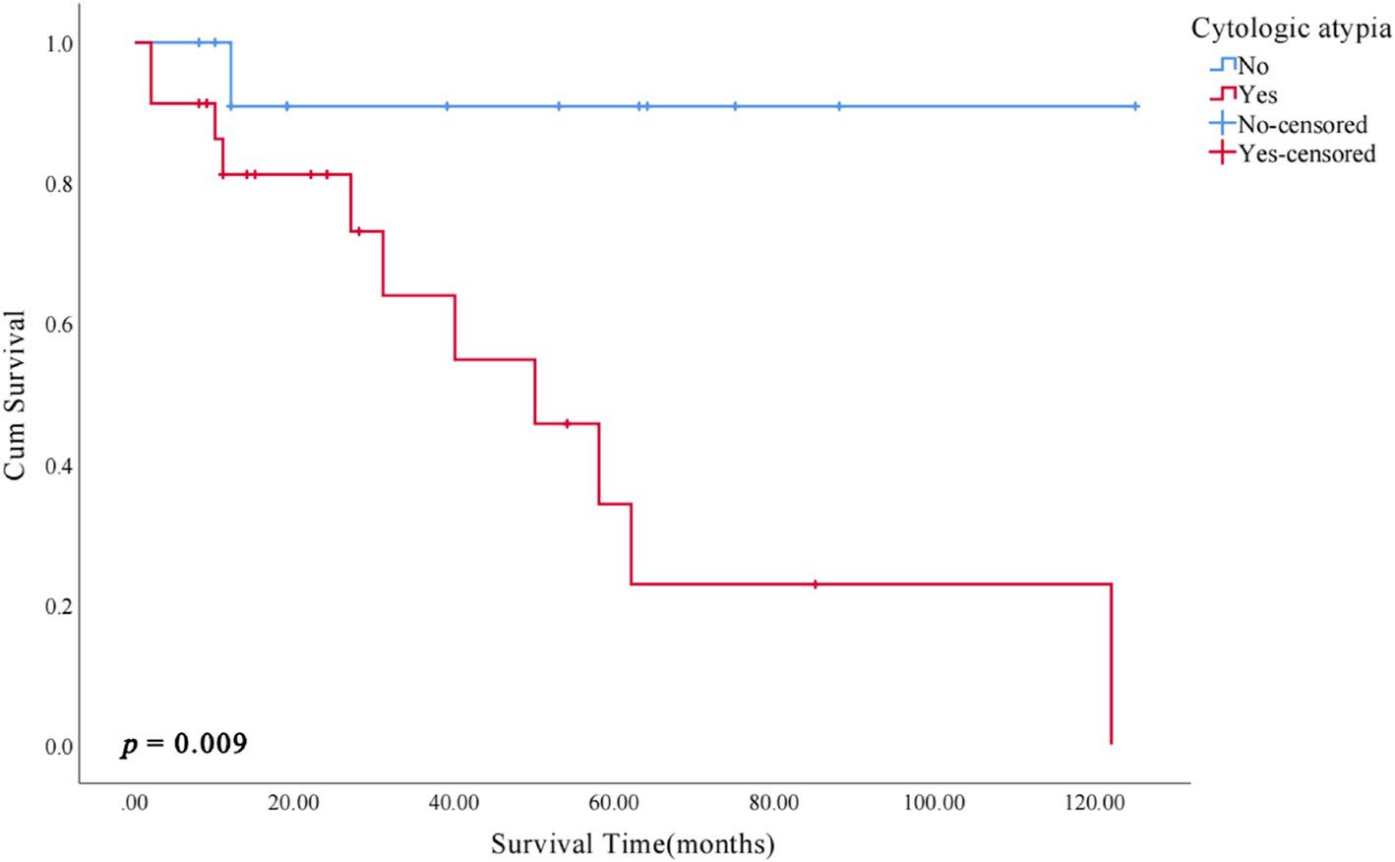
Kaplan–Meier malignant transformation curves according to cytologic atypia

**Fig. 6:**
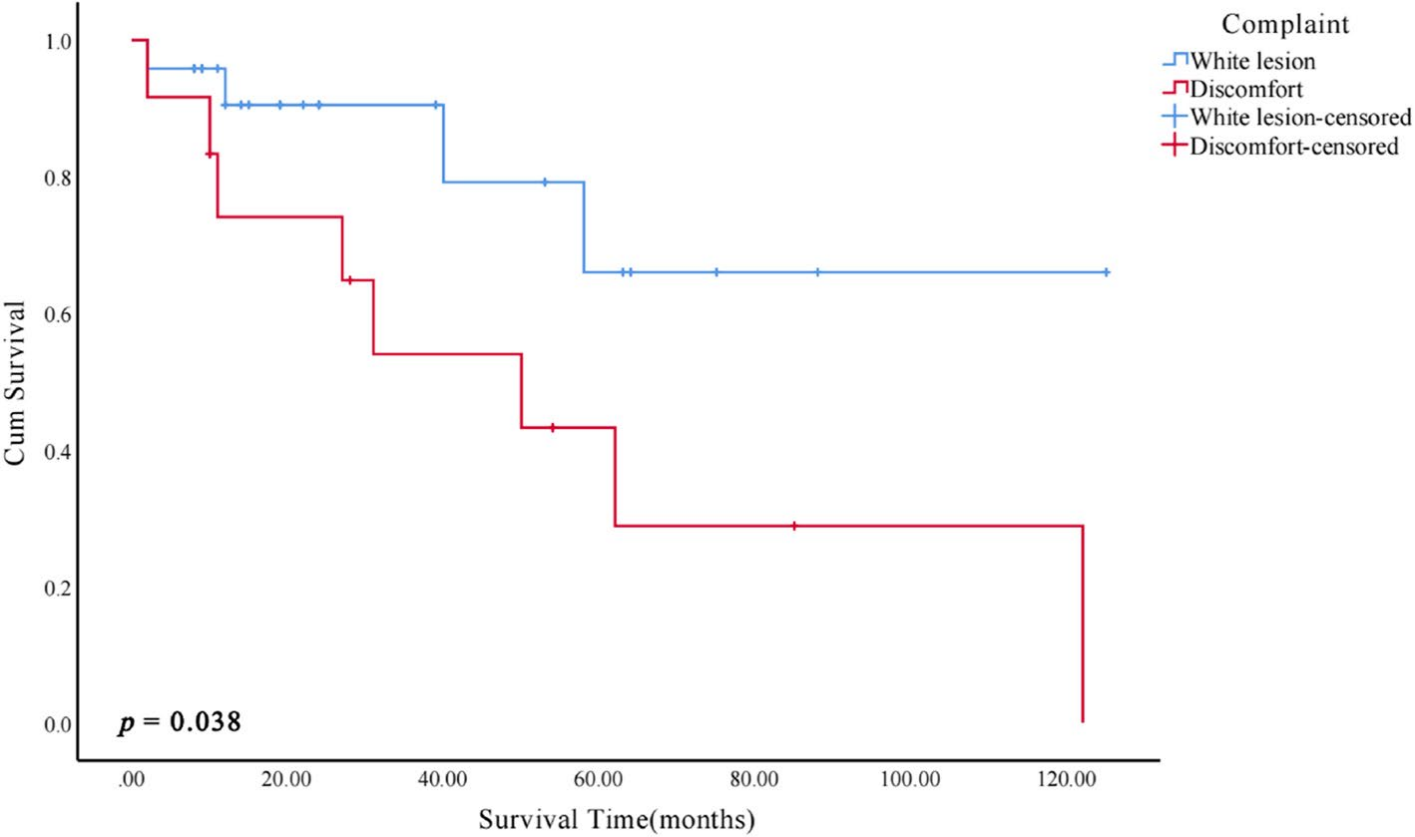
Kaplan–Meier malignant transformation curves according to complaint

**Fig. 7:**
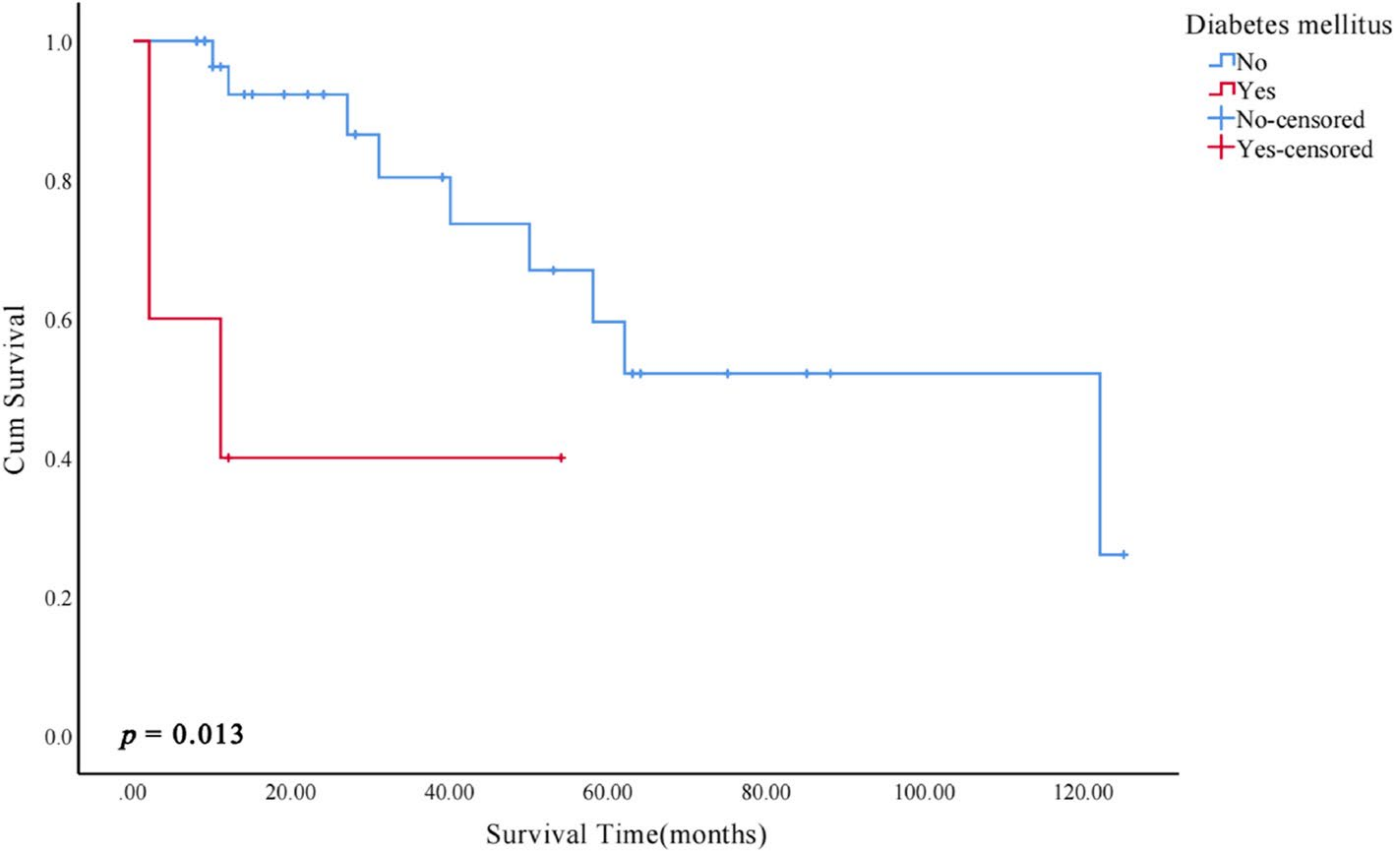
Kaplan–Meier malignant transformation curves according to diabetes mellitus

Kaplan–Meier survival analysis of clinicopathological features showed that patients with cytologic atypia, complaints of pain/rough/foreign body sensation, and diabetes mellitus had an increased risk of MT (*p* < 0.05, Figs. 5, 6 and 7).

## Discussion

Patients with indolent oral cancer can survive if appropriately treated early. However, public awareness is poor and many patients present with late-stage disease, contributing to a low survival rate [21]. PVL is characterized by a high rate of malignant transformation and persistence, making early diagnosis and appropriate treatment particularly crucial for improving patient survival rates.

Although PVL is considered a special type of leukoplakia, there are significant differences in clinical and pathological presentations between the two [22]. In the study of PVL, challenges include inconsistent diagnostic criteria and limited clinical pathological analysis. The malignancy rate of PVL correlates with the year of study [7]. To date, there are no recognized prognostic clinical pathological features or biomarkers for PVL malignancy [22, 23]. This study applies the most current diagnostic criteria to conduct a comprehensive and systematic analysis of the clinical pathological characteristics of PVL, calculates the malignancy rate, and investigates potential risk factors for MT.

In our study, the majority of patients were female, with a female-to-male ratio of 1.25. The average age at initial diagnosis in our patient cohort was 62.97 years, with female patients having an older average age(65.00 years) compared to males (60.44 years). This is consistent with prior studies, which revealed an overall female-to-male ratio of approximately 1.78, and the average age of patients is over 60 years [9]. Previous studies on oral leukoplakia have suggested that it predominantly affects the buccal mucosa or tongue, with smokers accounting for 42.5% to 59.2% of cases [24–26]. In contrast, PVL frequently affects the gingiva, and typically, over 80% of patients are non-smokers [12, 27]. In our study, a total of 77.22% of patients reported no history of smoking, consistent with previous findings that PVL frequently affected non-smokers. We found that 44.44% of PVL patients had their initial biopsy site on the gingiva, and the proportion of patients with gingiva involvement could reach 69.44% during the course. The average number of lesions per patient in our study was 3.36, closely matching the figure of 3.1 reported by Alkan et al. [14].

Previous studies on oral leukoplakia have suggested that over 40% showed no dysplasia at the initial biopsy [24–26]. In patients with PVL, prior studies have reported that about one-third of patients showed no epithelial dysplasia early on, with 16% possibly exhibiting moderate or severe dysplasia [10, 28, 29]. However, two studies have reported that over 50% of PVL patients had no dysplasia at the time of the initial biopsy [12, 27]. In our study, 22.22% (8/36) of patients showed no epithelial dysplasia, while 33.33% showed moderate to severe dysplasia at first biopsy, which may be related to the longer duration of the disease, later presentation for medical consultation, and progression of the disease.

Studies on the pathological characteristics of PVL are limited, generally focusing on the discussion of its pathological features and diagnostic criteria. Currently, there is no uniform standard for the pathological features and diagnostic criteria of PVL. Li et al. in 2021 described demarcated hyperkeratosis and "skip" segments in Proliferative Leukoplakia [30]. The 5th edition of the World Health Organization's head and neck tumor classification synthesized past research, indicating that early pathological manifestations in PVL additionally include premature keratinization, sharp lateral margins, and increased keratin [19]. Corrugated hyperortho or parakeratotic lesions with a verucco-papillary characteristic is considered a typical architecture in the early stage, without or with minimal cytologic atypia, while the description conducted subtle verbal difference in variable studies [28, 31]. Structural features in pathology are closely associated with progression and advanced lesions may develop an exophytic or endophytic growth pattern showing a verrucous, nodular, or bulky architecture with dysplasia [19, 28, 31].

Prior research suggested that early lesions in PVL patients rarely show cytologic atypia [19]. In our study, 63.87% of patients exhibited atypia at initial biopsy histopathology, which may be related to the widespread presence of epithelial dysplasia in 77.78% of patients at the initial biopsy [29, 32]. The corrugated hyperortho or parakeratotic lesions with a verucco-papillary characteristic were observed in almost all PVL patients (35/36). The verrucous, nodular, or bulky architecture occurred in 46.43% (13/28) patients with dysplasia, with a significant correlation (*p* = 0.001) to the presence of moderate or severe epithelial dysplasia, which is consistent with the current view that these are pathological manifestations in the progression of PVL [19, 28, 31]. The premature keratinization frequently occurred in patients with moderate or severe dysplasia. The previous study pointed out that the premature keratinization was associated with loss of heterozygosity in oral leukoplakia [33]. Whether such an association exists in PVL requires further research to verify.

PVL lesions are resistant to various treatments. We discovered 17 individuals relapsed among the 23 patients receiving treatment, which is roughly consistent with a meta-analysis indicating a recurrence rate of 67.2% [34]. Previous case studies have demonstrated that the use of topical photodynamic therapy with diode laser drilling pre-treatment can prevent the recurrence of lesions [35, 36]. However, the relationship between treatment methods and PVL malignancy transformation remains poorly researched [37]. In this study, the rate of malignant transformation in the treatment group(5/23) was lower than that in the untreated group(7/13), however, no statistically significant difference (*p* = 0.05). Limited sample size and follow-up durations as well as observation indicators may be reasons for this discrepancy. Large-scale randomized controlled trials are necessary to validate these findings in future research.

Risk factors for malignant transformation of oral leukoplakia include female gender, lesions located on the lateral or ventral tongue or floor of the mouth, lesions larger than 200mm^2^, and epithelial dysplasia [7, 25, 38]. The rate of malignant transformation in PVL substantially surpasses that of oral leukoplakia. Despite this, the literature on associated risk factors remains equivocal. A meta-analysis conducted previously has suggested that female patients with PVL are at an increased risk of developing oral cancer, particularly oral squamous cell carcinoma when compared to males [11]. In contrast, other studies have reported no significant correlation between gender and the risk of malignant transformation in PVL, which is in accord with the results of our analysis [9, 10, 12].

The gingiva is the most common site for MT in PVL patients, accounting for 28.95%-37.5% [10, 39]. In our study, this proportion is 33.33%. Malignant transformation in PVL may occur in areas neighboring the biopsy site or in two separate, distant locations, underscoring the importance of monitoring all lesions in the patient. Previous investigations have suggested that a larger lesion area indicates an increased risk of malignant transformation in oral leukoplakia and may be more advanced with a greater challenge for excision in PVL [12, 40, 41]. Cerero-Lapiedra et al. identified the lesion exceeding 3.0 cm as a minor criterion of PVL [13]. We found the median maximum diameter of lesions in the malignant group was 2.50 cm, exceeding that of the non-malignant group, which measured 2.10 cm. The difference lacked statistical significance.

Our study found a significant correlation (*p* < 0.05) between the presence of cytologic atypia and the risk of malignant transformation, indicating that cytologic atypia should not be overlooked in the prognostic study of PVL. Previous research has shown that cellular morphology and nuclear shape can be used for the early identification of oral cancer [42, 43]. In patients with oral epithelial dysplasia, abnormal cell size, abnormal cell morphology, abnormal nuclear morphology, nuclear enlargement, and increased number of nuclei are associated with the risk of MT [44–46].

In the survival analysis, we found that patients with diabetes may take a higher risk of malignant transformation (Log-rank, *p* = 0.013). A previous study at our institution discovered that hyperglycemia significantly increased the risk of malignant transformation in oral leukoplakia [47]. However, since there were only 5 patients with diabetes in this study, the analysis results should be interpreted with caution.

At present, the sample size of studies examining the clinical characteristics of PVL ranges from 3 to 80, with more than half having a sample size of less than 30. The majority of these reports are concentrated in European and American countries, and there is a paucity of data from Asian populations. The total sample size across three studies from Asian population sources amounts to only 22, with follow-up times ranging from 3.3 to 16 years [9–11].

However, this study has several limitations: firstly, the total sample size was 36, which necessitates cautious interpretation of statistical differences. Additionally, as this was a retrospective study, the number and duration of follow-up visits varied among patients, and no comparative analysis was conducted on the clinical and pathological changes observed during different follow-up visits the analysis results should be interpreted with caution. Regardless, the findings of this study further contribute to the database of Asian patients with PVL.

## Conclusion

Proliferative verrucous leukoplakia is more common in elderly women and typically occurs in the gingiva. Most patients report no discomfort and have no history of smoking; nevertheless, the rate of malignant transformation can reach 33.33%. Currently, there is a lack of consensus on the factors associated with the risk of malignant transformation. The presence of symptoms such as pain, roughness, foreign body sensation, and specific pathological features may indicate an increased risk of malignancy. Further research is required to elucidate the impact of these clinicopathological features on the risk of malignant transformation, especially in large-scale cohort studies with prolonged follow-up durations.

## Data Availability

The datasets generated and analyzed during the current study are not publicly available due to the need to maintain patient confidentiality. However, they can be obtained from the corresponding author upon reasonable request.
